# Preventive Effect of a Synbiotic Combination of Galacto- and Fructooligosaccharides Mixture With *Bifidobacterium breve* M-16V in a Model of Multiple Rotavirus Infections

**DOI:** 10.3389/fimmu.2018.01318

**Published:** 2018-06-11

**Authors:** Maria del Mar Rigo-Adrover, Kees van Limpt, Karen Knipping, Johan Garssen, Jan Knol, Adele Costabile, Àngels Franch, Margarida Castell, Francisco José Pérez-Cano

**Affiliations:** ^1^Departament de Bioquímica i Fisiologia, Facultat de Farmàcia i Ciències de l’Alimentació, University of Barcelona (UB), Barcelona, Spain; ^2^Institut de Recerca en Nutrició i Seguretat Alimentària (INSA), University of Barcelona (UB), Santa Coloma de Gramanet, Spain; ^3^Nutricia Research, Utrecht, Netherlands; ^4^Division of Pharmacology, Faculty of Science, Utrecht Institute for Pharmaceutical Sciences, Utrecht University, Utrecht, Netherlands; ^5^Health Sciences Research Centre, Life Science Department, Whitelands College, University of Roehampton, London, United Kingdom

**Keywords:** prebiotic, probiotic, synbiotic, rotavirus, FOS, GOS, *Bifidobacterium breve*

## Abstract

Rotavirus (RV) causes morbidity and mortality among infants worldwide, and there is evidence that probiotics and prebiotics can have a positive influence against infective processes such as that due to RV. The aim of this study was to evidence a preventive role of one prebiotic mixture (of short-chain galactooligosaccharide/long-chain fructooligosaccharide), the probiotic *Bifidobacterium breve* M-16V and the combination of the prebiotic and the probiotic, as a synbiotic, in a suckling rat double-RV infection model. Hyperimmune bovine colostrum was used as protection control. The first infection was induced with RV SA11 and the second one with EDIM. Clinical variables and immune response were evaluated after both infections. Dietary interventions ameliorated clinical symptoms after the first infection. The prebiotic and the synbiotic significantly reduced viral shedding after the first infection, but all the interventions showed higher viral load than in the RV group after the second infection. All interventions modulated *ex vivo* antibody and cytokine production, gut wash cytokine levels and small intestine gene expression after both infections. In conclusion, a daily supplement of the products tested in this preclinical model is highly effective in preventing RV-induced diarrhea but allowing the boost of the early immune response for a future immune response against reinfection, suggesting that these components may be potential agents for modulating RV infection in infants.

## Introduction

Rotavirus (RV) is the most common etiological agent of severe dehydrating diarrhea among children under the age of 5 worldwide (1). The global median RV detection proportion in hospitalized children <5 years of age with acute gastroenteritis (diarrhea) caused by RV was of about 38% in 2012 (2), whereas a range of about 215,000 deaths in 2013 were estimated in this context (3). The main symptoms are fever, vomiting, and diarrhea in children, for 3–8 days (4, 5). Virtually every child in the world will be infected with RV in the first 3 years of life (6), and several reinfections usually occur, although they tend to be less severe than the first infection (5). RV belongs to the *Reoviridae* family and is a non-enveloped, icosahedral, double-stranded RNA covered by a triple layer of capsids. Its viral genome encodes for six structural (VPs) and six non-structural proteins (4, 7–9). RVs are classified in groups (A–G), subgroups (I and II) and serotypes/genotypes (based on the antigenic differences among the external capsid). RVs from group A are the main human pathogens, and their transmission is fecal-oral, with a higher prevalence in the winter season. They infect mature enterocytes of the small intestine, but the entry mechanism and pathogenesis is still not well known (10, 11).

After RV infection, the innate and adaptive immune responses are induced and will lead to the production of cytokines and RV-specific antibodies (12, 13). Natural killer lymphocytes and dendritic cells are crucial for response activation of both innate and adaptive immunity (14). Besides T-cell action on infected cells and cytokine production, antibody production by B-cells at local and systemic level is required for long-term protection, especially IgA (15–18). However, immunity after an RV infection is not fully complete and reinfections usually occur, although they are less severe than the first infection (5).

Several interventional approaches can be addressed because oral rehydration, the most common treatment (19, 20) is not sufficient and immune modulatory interventions such as vaccination or dietary supplementation with active agents need to be included. In this regard, two live attenuated oral vaccines, Rotateq (Merck & Co., PA, USA) and Rotarix (GSK Biologicals, Rixensart, Belgium), have been available since 2006. RV vaccines have demonstrated safety and efficacy (2, 3, 7, 21), but their implementation is not yet global (6, 20). With regard to nutritional interventions, bioactive components from breast milk, which are found in high proportions in colostrum, probiotics or prebiotics are the leading studied products.

Thus far, whey protein concentrates and bovine colostrum (with or without RV-specific antibodies) have shown effective protection against RV disease (22–28), and thus are candidates for protection control in dietary interventional studies. Moreover, a colostrum from cows immunized with a RV strain and therefore with high anti-RV-Ab titers [hyperimmune bovine colostrum (HBC)] has also been tested in different RV-infection models with very satisfactory results (25, 26).

Probiotics are live microorganisms that, when administered in adequate amounts, confer a health benefit on the host (29). Several species of the *Lactobacillus* and *Bifidobacterium* genera have been studied and have demonstrated some positive effects against RV infection by modulating several mechanisms: chloride secretion and oxidative stress (30), virus replication and adhesion capacity (31, 32), and host immune cell response (33). Due to these actions, probiotics have led to clinical amelioration in animal models (34, 35), as well as some clinical and immunological benefits in RV-infected babies in clinical trials (36–39). On the other hand, prebiotics are indigestible food ingredients that reach the colon and promote the growth or activity of certain beneficial species in the intestinal microbiota, thereby generating a health benefit (40, 41). Besides their beneficial role in modulating short-chain fatty acids (SCFAs) or even intestinal IgA secretion (41), only a few interventional studies have been conducted to investigate the role of prebiotics against RV infection. However, because of their importance in early life, such as through human milk oligosaccharides or specific mixtures of short-chain galactooligosaccharides (scGOSs) and long-chain fructooligosaccharides (lcFOSs), which are widely used in infant formulas, prebiotics have been studied most in both preclinical (42, 43) and clinical (44, 45) studies involving RV infection.

In previous studies, the effectiveness of a prebiotic mixture, scGOS/lcFOS, in a proportion of 9:1 (Nutricia Research, The Netherlands), with or without the probiotic *Bifidobacterium breve* M-16V, and the probiotic *B. breve* M-16V alone, has been tested in an RV-infected neonatal rat model (43). However, one of the limitations of these studies was that the frequency of reinfection in humans is very high, and the impact of these active compounds on the host immunity against a new RV infective process is not known. A double-infection model will provide significant information on how the protection against the first infection due to the prebiotic or probiotic interventions will affect the second infection, mainly in terms of immune response. For that reason, a rat double-RV infection model has been used to study this type of effect due to interventional approaches in early life.

In light of the above comments, the hypothesis of this study is that the modulation of first infection by microbial modulator components may regulate the onset of a future reinfection. Thus, the aim of this study was to test the effectiveness of a prebiotic mixture, scGOS/lcFOS (9:1), with or without the probiotic *B. breve* M-16V, and the probiotic *B. breve* M-16V alone, in a double-RV infection neonatal rat model.

## Materials and Methods

### Animals

Twenty-four G14 pregnant Lewis rats (LEW/Han^®^Hsd) were obtained from Harlan (Horst, The Netherlands). They were housed individually in cages (2184L Eurostandard Type II L, Tecniplast, West Chester, PA, USA) with large fibrous particles bedding and tissue papers as enrichment and, monitored daily and allowed to deliver at term. The day of birth was registered as day 1 of life. Litters were unified to seven pups per lactating dam with similar number of each sex in each nest. The pups had free access to the nipples (until PN16) and the rat diet. The animals were housed in controlled temperature and humidity conditions, in a 12:12 h light/dark cycle. They were located in a special safe, isolated room at the Animal Service of the Faculty of Pharmacy, University of Barcelona, designed and authorized for working under biosecurity level 2 conditions. The dams were fed with a commercial diet (Teklad Global Diet 2014, Envigo, Indianapolis, IN, USA) corresponding to the American Institute of Nutrition 93G formulation (46) and given water *ad libitum*.

All experimental procedures were conducted in accordance with the institutional guidelines for the care and use of laboratory animals and were approved by the Ethical Committee for Animal Experimentation of the University of Barcelona and the Catalonia Government (CEEA-UB Ref. 493/12, DAAM: 6905), in full compliance to national legislation following the EU-Directive 2010/63/EU for the protection of animals used for scientific purposes.

### Viruses

Two different type A viruses were used for the experiments: simian agent 11 (SA11) and the epizootic diarrhea of infant mouse virus (EDIM). The virus selected for the first infection was the SA11, an RV strain produced by the “Enteric Virus Group” of the University of Barcelona, as used in previous studies (23, 43, 47). The virus selected for the second infection was the EDIM, obtained *in vivo* from inoculated neonatal BALB/c mice (Janvier, La Plaine Saint Denis Cedex, France) with an initial inoculum of the virus (Nutricia Research, the Netherlands). Briefly, stool samples were collected twice a day from day 3 to day 13, pooled and homogenized by using Polytron^®^ (Kinematica, Luzern, Switzerland). EDIM was extracted with Genetron^®^ (1,1,2-trichloro-1,2,2-trifluoroethane, Sigma-Aldrich, Madrid, Spain) and later quantified by ELISA as described in previous studies (26).

### Experimental Design and Dietary Supplementation

Suckling rats were distributed into following six different experimental groups: reference (REF), double rotavirus infection (DRI), hyperimmune bovine colostrum (HBC), prebiotic (PRE), probiotic (PRO), and synbiotic (SYN).

Each group was composed of three litters of seven pups each (*n* = 21/group). The dietary intervention was orally administered to the animals as previously described (48), using low-capacity syringes (Hamilton Bonaduz, Bonaduz, Switzerland) adapted to 25- or 23-caliber forced alimentation tubes, 27 mm in length (ASICO, Westmont, IL, USA), with the different products (HBC, PRE, PRO, and SYN groups) or vehicles (DRI and REF groups) from day 3 until day 14 of life. The HBC group received 50 mg/animal/day of “anti-RV hyperimmune bovine colostrum”; this HBC was tittered to be effective in blocking the virus *in vitro* in concentrations higher than 10 µg/mL (gently given by Dr. Viviana Parreño, Institute of Virology, CICV and A-INTA, Castelar, Argentina). The PRE supplement consisted of a combination of scGOS and lcFOS, in a 9:1 ratio (Nutricia, The Netherlands), and was administered in a dose of 0.8/100 g of body weight/day. The PRO group received *B. breve* M-16V (Morinaga Milk Industry Co., Ltd., Tokyo, Japan) suspension in a dose of 4.5 × 10^8^ UFC/100 g of body weight/day. The SYN group received both PRE and PRO products in the same concentrations as when administered alone. A group of rats receiving bottled mineral water as vehicle was the inoculated control group (DRI group), while another group receiving water acted as the non-inoculated control group (REF group).

The RV inoculations were carried out in all the experimental groups, with the exception of the REF group, as previously described (47). They were done 1 h after separation from their dams to avoid interference between RV and milk components following previous procedures from the group (23, 43, 47). This action is required due to the presence of human breast milk components that can block the virus and reduce its infection such as oligosaccharides or maternal antibodies (49). SA11 was selected as the first infective virus because previous studies allowed us to obtain a rat model of mild diarrhea in early life (23, 43, 47). It was orally inoculated at day 6 of life in a dose of ~2 × 10^8^ TCID_50_ RV/rat in 100 µL of phosphate-buffered solution (PBS). EDIM was used as the second infective virus, inoculated at day 17 in a dose of ~1.3 × 10^8^ RV/mL in 100 µL, when the intestinal immune system was still in maturation (50). Furthermore, because of the importance of the bioactive factors present in maternal milk in protecting the pups from infection, the weaning day was on day 16, to induce a lower defensive situation in the pups. In addition, there were two more groups: those infected singly either with SA11 (SA11) or with EDIM. Moreover, selected animals from the supplemented groups were not submitted to the second infection with EDIM and therefore preliminary data from animals infected singly with SA11 and with dietary interventions (SA11 + HBC, SA11 + PRE, SA11 + PRO, and SA11 + SYN) were also obtained at the end of the study.

Clinical evaluation was performed daily from the first day of supplementation (day 3) until the end of the study (day 28). One-third of the animals from each group were euthanized on day 16 of life, and the rest on day 28. Fecal samples were collected daily during the study, and blood, small intestine, intestinal wash samples, small intestinal tissue and isolated spleen, and mesenteric lymph node (MLN) cells at the end point. Body temperature and fecal pH were measured during the peri-inoculation period of both infections. The delayed-type hypersensitivity (DTH) response was determined at the end of the study.

The animals were weighed and monitored daily during the early light phase to obtain data regarding the influence of the virus inoculation, clinical development and nutritional intervention on growth and fecal features. This was done after separation of the pups from their mother, during the handling and before oral administration; the whole litter together was reunited with the dam after interventional actions.

### Clinical Indexes and Fecal Specimen Collection

SA11 and EDIM infections were evaluated from day 2 to day 28 of life by the growth rate and clinical indexes that require daily fecal sampling as previously described (47). Briefly, fecal sampling was performed once a day by gently pressing and massaging the abdomen. Specimens were immediately scored for severity of diarrhea, weighed and frozen at −20°C for further analysis. The severity of diarrhea was expressed by the fecal weight and by scoring stools from 1 to 4 [diarrhea index (DI)] based on color, texture and amount as follows: normal (DI = 1), loose yellow-green (DI = 2), totally loose yellow-green (DI = 3), and high amount of watery (DI = 4) feces. Diarrhea scores ≥ 2 indicate diarrheic feces whereas scores of DI < 2 indicate absence of diarrhea. The area under the curve of severity (sAUC) during 0–6 DPI was calculated as a global value of severity. The maximum diarrhea index (MDI) was defined as the highest score during the diarrhea period (DP). Incidence of diarrhea was expressed by the percentage of diarrheic animals (%DA, consisting of the percentage of diarrheic animals taking into account the number of animals in each group) and by the percentage of diarrheic feces (%DF, consisting of the percentage of diarrheic samples taking into consideration the number of total samples collected every day in each group). The AUCs of %DA and %DF (daAUC and dfAUC) during 0–6 DPI were calculated as a global value of incidence. The AUCs for severity, %DA and %DF were also calculated taking into account the basal values due to intrinsic aspects of each treatment (normalized AUC). The maximum percentages of diarrheic animals (MDA) and diarrheic feces (MDF) were defined as the highest values during the DP. The days when MDI, MDA, and MDF were achieved were also used as indicators, called MDId, MDAd, and MDFd, respectively. The DP was calculated for each animal as the interval between the first [day of diarrhea beginning (DDB)] and last [day of diarrhea ending (DDE)] day of diarrhea. The actual days with diarrhea (DwD) within the DP were also counted (DwD).

### Fecal pH Determination

Fecal samples from the peri-inoculation period of the virus were diluted in distilled water (up to 200 mg/mL) and gently agitated. Their pH was measured using a 5207 pH electrode for surfaces (Crison Instruments, Barcelona, Spain) and a micropH 2001 pH meter (Crison Instruments).

### Body Temperature Determination

The rats’ body temperature was measured with a TEMP JKT thermometer (Oakton, Vernon Hills, IL, USA) and an RET-3-ISO rectal probe for neonatal rats (Physitemp, Clifton, NJ, USA) and with the aid of peanut oil (Acofarma, Terrassa, Spain) to lubricate. This measure was taken 1–2 days before the inoculation of the virus (initial temperature) and 1–2 days after inoculation of the virus (final temperature). Results were expressed as the absolute increase in temperature taking into account the final temperature (after RV inoculation) with respect to the initial temperature (before RV inoculation).

### Viral Shedding

Fecal samples from selected days of interest were diluted in PBS (10 mg/mL) and homogenized using a FastPrep (MP Biomedicals, Santa Ana, CA, USA). Homogenates were centrifuged (19,000 *g*, 3 min), and supernatants were frozen at −20°C until use. SA11 and EDIM particles in fecal samples were quantified by ELISA, as previously described (47). Titrated dilutions of inactivated SA11 virus particles, ranging from 4 × 10^5^ to 2.5 × 10^4^/mL, were used as standard in each plate.

### DTH Response

Two days before sacrifice (day 26), the thickness of both the right and left ears of every animal was measured to constitute the basal conditions by using a 7309 pocket thickness gauge (Mituyoto, Hampshire, UK). For virus priming, animals were anesthetized with isoflurane (Abbott Laboratories, Berkshire, UK), and the virus was injected into the ear using low-volume Hamilton syringes (100 µL) connected to needles (30 G 1/2 0.3 × 13). A volume of 20 µL of UV-inactivated virus (~0.5 × 10^6^ RV particles/mL) was injected into the right ear (RE), and the same volume of PBS was injected into the left ear (LE). After 24 and 48 h, before sacrifice, an evaluation of the ear thickness was performed again. Results are expressed as the increase in thickness of the RE minus the increase in thickness of the LE (to eliminate the intrinsic increase due to the puncture).

### Blood, Spleen, and Intestinal Sample Collection

After previous anesthesia with intramuscular ketamine/xylazine injection (Imalgene 100 mg/mL, Merial Laboratorios, Barcelona, Spain/Rompun^®^ 20 mg/mL, Bayer Hispania, Sant Joan Despí, Spain), rats from each group were euthanized at days 16 or 28 by opening the peritoneal cavity and disrupting the diaphragm. Blood was collected by cardiac puncture, and after centrifugation sera was stored at −20°C until analysis. The small intestine was weighed, cut into 5 mm pieces and incubated with PBS for 10 min at 37°C in a shaker to obtain the gut wash (GW). After centrifugation, supernatants were stored at −80°C until analysis. One centimeter of tissue from the middle of the small intestine was obtained and kept in RNAlater from Ambion (Thermo Fisher Scientific, Barcelona, Spain) at −20°C for further determinations. The spleen and MLNs were removed under sterile conditions. Spleen and MLN cells were isolated as previously described (51). Cell numbers and viability were determined using an automated cell counter after staining dead cells with trypan blue (Countess™, Invitrogen, Madrid, Spain). Isolated mononuclear cells from the spleen and MLNs were cultured for 72 h under SA11/EDIM mix stimulatory conditions (10^5^ viral particles/mL). After incubation, the 24-well plate was centrifuged, and the supernatants were collected and kept at −80°C until cytokine determination.

### ELISA for Total IgA and IgM Antibody Quantification in Serum and GW

Ninety-six-well plates (Nunc MaxiSorp, Wiesbaden, Germany) were coated with purified mouse anti-rat IgA or IgM (BD Biosciences, Heidelberg, Germany). After blocking with PBS-1% bovine serum albumin [BSA, 1 h, room temperature (RT)], appropriate diluted sera or intestinal wash samples were added (3 h, RT). After washing, biotin anti-rat IgA or IgM from BD Biosciences, followed by peroxidase-conjugated extravidin (Sigma-Aldrich), were added. Subsequently, substrate was added, as previously described (47). Dilutions of purified rat IgA or IgM (BD Biosciences) ranging from 80 to 1.25 ng/mL were used as a standard in each plate. Quadratic polynomial adjustment was used.

### ELISA for Specific Anti-RV Total and IgM Antibody Quantification in Serum and GW

Ninety-six-well plates (Nunc MaxiSorp) were coated with UV-inactivated SA11 particles at 10^5^/mL. After blocking with PBS-1% BSA (1 h, RT), appropriate diluted sera or intestinal wash samples were added (3 h, RT). After washing, rabbit anti-rat Ig conjugated to peroxidase from Dako (Barcelona, Spain) or mouse biotinylated anti-rat IgM (G53-238) monoclonal antibody from BD Biosciences, followed by peroxidase-conjugated extravidin (Sigma-Aldrich), were added. Subsequently, substrate was added, as previously described (47). Pooled sera from dams of inoculated litters were used as a standard in each plate. Quadratic polynomial adjustment was used.

### ELISPOT for Specific Anti-RV Antibody Production

An ELISPOT technique was used to quantify anti-RV Ig-secreting cells (SC) from spleen and MLN by following previous experience (52). Ninety-six-well nitrocellulose plates (Merck Millipore) were coated with viral particles of SA11 or EDIM (10^5^ particles/mL) in sterile conditions (overnight, 4°C). The remaining binding sites were blocked with complete media for 1 h at 37°C. Freshly isolated cells were plated at serial dilutions (2 × 10^5^, 1 × 10^5^, 0.5 × 10^5^, and 0.25 × 10^5^ cells/well) and incubated for 3 days (37°C, 5% CO_2_). Cells were then removed by washing 10 times with PBS containing 0.25% of Tween 20, and once with distilled water. Biotin-conjugated anti-rat IgM Ab (BD Pharmingen, San Diego, CA, USA, 2 mg/L in PBS) was added and incubated for 2 h. The plate was washed again, and then incubated with extrAvidin-peroxidase conjugate (Sigma-Aldrich, 4 mg/L) for 1 h. Spots, each one corresponding to one anti-RV Ig-SC, were visualized after adding the substrate solution (3-amino-9-ethyl-carbazole plus H_2_O_2_ in 0.1 mol/L acetate solution). The reaction was stopped using tap water. Spots were counted automatically by computer-assisted ELISPOT image analysis (ELISPOT reader system, AID, Strasberg, Germany) and expressed as the number of Ig-SC per 10^6^ cells.

### Bead Immunoassay

The cytokine concentration in supernatants from stimulated spleen cells and from GW samples was measured. Antiviral and Th1 (IFNγ), Th2 (IL-4), anti-inflammatory and regulatory (IL-10), and pro-inflammatory (TNFα) cytokines were evaluated. Molecule determinations were performed using a BD™ Cytometric Bead Assay Rat Soluble Protein Flex Set (BD Biosciences, Madrid, Spain) as detailed in previous studies (53). A FacsAria SORP sorter (BD, San José, CA, USA) from the cytometry service of the Scientific and Technological Centers of the University of Barcelona (CCiT-UB) was used. Data analysis was performed using the FlowJo 10.0.7 software (Tree Star, Inc., Ashland, OR, USA).

### Real-Time PCR for Small Intestine Gene Expression

Gene expression of different genes that could be of interest was evaluated. Specifically, we selected target genes expressed in the small intestine at day 28 that could be representative of the possible effect that the dietary intervention could modulate: receptors from the TLR family (i.e., TLR2 and TLR4), Th1 and Th2 responses (IFNγ and IL-4, respectively), molecules involved in the Tight Junction (TJ, i.e., Occludin, Claudin-2), innate defenses (mucin), and regulatory and tolerogenic response (IL-10, TGF-β, and FoxP3).

On sacrifice day, the intestinal tissue was obtained and kept in RNAlater from Ambion (Thermo Fisher Scientific) at −20°C until analysis. Homogenization was performed by using lysing matrix tubes (MP Biomedicals, Illkirch, France) in a FastPrep-24 instrument (MP Biomedicals). RNA was isolated with the RNeasy^®^ Mini Kit (Qiagen, Madrid, Spain) following the manufacturer’s instructions as previously described (54). A Nanodrop spectrophotometer and Nanodrop IVD-1000 v.3.1.2 software (Nanodrop Technologies, Wilmington, DE, USA) were used to quantify the amount and purity of RNA obtained. The RNA integrity number was evaluated, using the Genomic Service of the SCT-UB, to ensure the quality and integrity of the material. RNA was reverse-transcribed, as previously described (54). PCR Taqman^®^ primers and probes specific for rat target genes and HPRT as endogenous control were used (Assays on Demand™, Gene Expression Products, AB). The amount of target mRNA, normalized with an endogenous control (HPRT) and relative to a calibrator (tissue samples from the REF group as the control group), was given by the 2^−ΔΔCt^ method, as previously described (54). Results are expressed as the mean ± SEM of the percentage of these values for each experimental group compared with its reference group, which represents 100% of gene expression.

### Short-Chain Fatty Acids

The production of acetic, propionic, formic, lactic, and butyric acids in the fecal samples were determined by HPLC (Merck) equipped with RI detection. The column used was an ion-exclusion REZEX-ROA organic acid column (Phenomenex Inc., UK), and temperature maintained at 84°C. Sulfuric acid in HPLC-grade H_2_O (0.0025 mmol/L) was used as the eluent, and the flow rate was maintained at 0.5 mL/min. Aliquots of 300 mg (w/v) of feces collected in microcentrifuge tubes were centrifuged at 13,000 *g* for 10 min to remove all particulate matter. The fecal supernatants were then filtered using 0.22 µm low protein binding Durapore polyvianylidene fluoride membranes (Millex; EMD Millipore, Billerica, MA, USA) to remove possible bacteria. 50 µL of each sample was injected with a run of 45 min into HPLC. Peaks were integrated using the Atlas Lab managing software (Thermo Lab Systems, Mainz, Germany). Quantification of the samples was obtained through calibration curves of acetic, propionic, formic, lactic, and butyric acids in concentrations ranging between 12.5 and 100 mM.

### Statistical Analysis

Taking into account the number of pups as statistical unit, the number of animals used in each group for detecting a statistically significant difference among groups assuming that there is no dropout rate and type I error of 0.05 (two-sided) was calculated by the Appraising Project Office’s program from the Universidad Miguel Hernández de Elche (Alicante). The variables used for the calculation included both the clinical outcomes, particularly the severity score, and immune variables, by means of the anti-RV Ig levels. Moreover, as previous studies demonstrated the importance of the litter effect (23, 43, 47), independently of the number of animals obtained above, at least three litters were required for each group. Taking this into account, three litters of seven animals per group were enough for the sample-size estimation performed. The final number of animals was not affected by the dropouts or outliers, which did not occur in this study.

The PASW Statistics 22 software package (SPSS Inc., Chicago, IL, USA) was used for the statistical analysis. The Kolmogorov–Smirnov test was applied to assess normal distribution, followed by Levene’s test to determine variance equality. A conventional one-way ANOVA test was performed taking the experimental group as the independent variable. When virus inoculation or dietary interventions had a significant effect on the dependent variable, Scheffé’s *post hoc* test was applied. The Kruskal–Wallis and Mann–Whitney *U* tests were used when non-normal distribution or different variance was found. Finally, the chi-square test was used to compare frequencies. Differences were considered significant at *p* values of < 0.05. All the results are expressed as mean ± SEM of a certain number of animals.

## Results

### Body Weight

**Figure 1:**
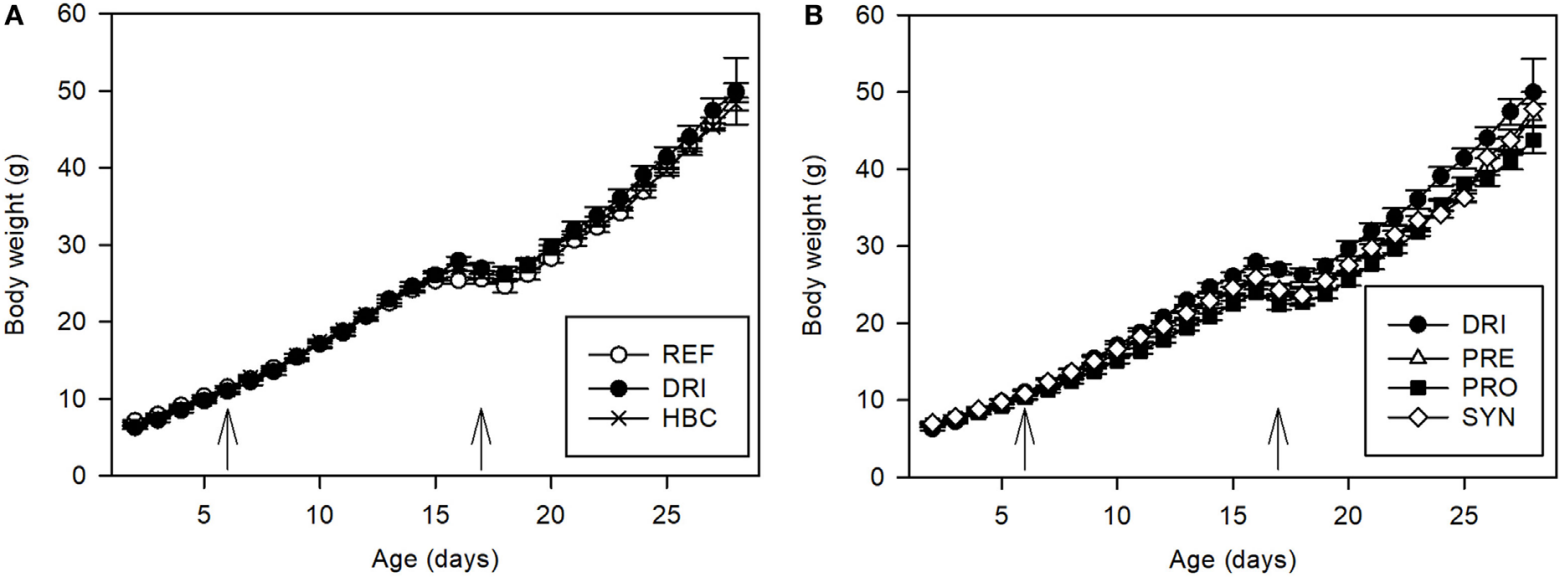
Body weight (g) during the study, before, and after the virus inoculations (indicated by arrows) on day 6 and day 17 **(A)** for REF, DRI, and HBC groups; **(B)** for DRI, PRE, PRO, and SYN groups. Results are expressed as mean values ± SEM. Statistical significance is explained in the text (*n* = 12–21 animals/group). Groups: REF, reference; DRI, double rotavirus infected; HBC, hyperimmune bovine colostrum; PRE, prebiotic; PRO, probiotic; SYN, synbiotic.

Body weight was recorded between days 2 and 28 of life. The former was about 6–7 g and the latter about 44–49 g. As can be seen in Figure 1, the inoculation with the first virus (SA11) was performed on pups with a body weight of around 10–11 g, and it had no impact on this variable. With regard to the second inoculation with EDIM, a weight loss was observed on day 17 (*p* < 0.01). However, it cannot be ascribed to the infection but is due to the early weaning, since the REF group showed the same pattern. The HBC intervention did not induce changes in the body weight course compared with the REF and DRI groups (Figure 1A).

As regards the effect of dietary supplementation on body weight, Figure 1B shows that the prebiotic intervention (PRE) induced a slight decrease in the weight of the animals before the first inoculation (first week of life) when compared with the REF group (*p* < 0.05). The SA11 inoculation did not affect the growth of these animals in the next few days (when compared with REF or DRI) because the pattern was similar to before. After day 13, the prebiotic group also started to weigh less than the DRI group (*p* < 0.05). The EDIM inoculation did not affect this variable either, but the early weaning did cause a loss of body weight as in the REF and DRI groups.

The dietary supplementation with the PRO and the SYN (Figure 1B) induced a pattern of body weight similar to that found for the prebiotic administration. During life, the animals from the PRO group had a lower body weight than those from the REF and the DRI groups (*p* < 0.05), although this difference was not so evident for the animals in the SYN group. The same body weight loss after early weaning (day 17) was again observed, but there was no effect due to SA11 or EDIM inoculation.

When the global behavior during the post-infective period was studied after the first RV inoculation, the percentage of body weight increase between day 7 and day 17 in all groups was between 123 and 155%, with all the inoculated animals’ values being higher than those from the REF group (*p* < 0.05). After the second infection with EDIM (day 17–27), no differences among groups were found.

### Incidence of Diarrhea

**Figure 2:**
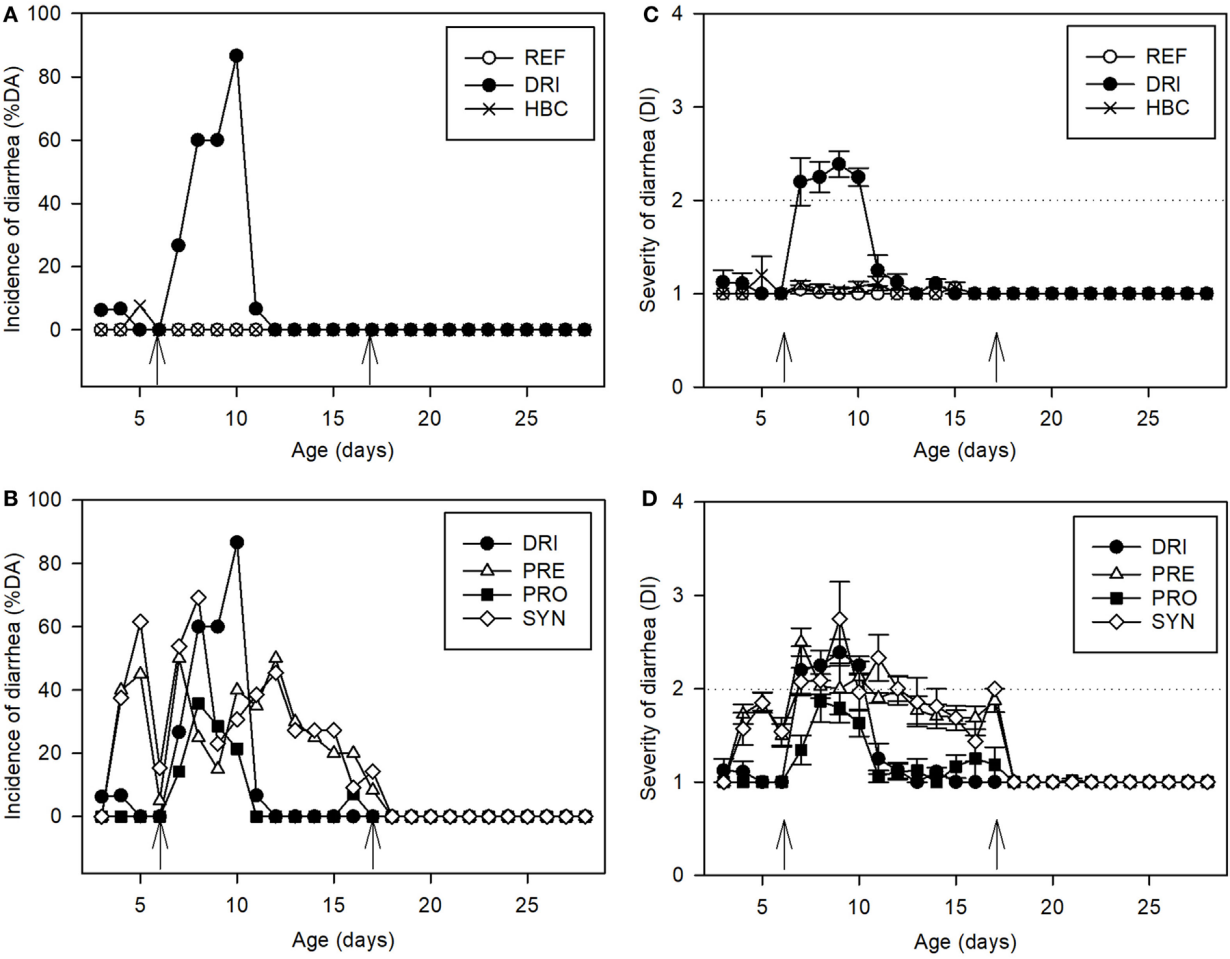
Clinical indexes. Incidence of diarrhea **(A)** for REF, DRI, and HBC groups; **(B)** for DRI, PRE, PRO, and SYN groups. Results are expressed as % of diarrheic animals. Severity of diarrhea **(C)** for REF, DRI, and HBC groups; **(D)** for DRI, PRE, PRO, and SYN groups: fecal samples are scored from 1 to 4 based on color, texture, and amount of stool. Scores on diarrhea index ≥ 2 indicate diarrheic feces. The inoculation days are indicated by arrows. Results are expressed as mean ± SEM. Statistical differences mentioned in the text (*n* = 12–21 values/group). Groups: REF, reference; DRI, double rotavirus infected; HBC, hyperimmune bovine colostrum; PRE, prebiotic; PRO, probiotic; SYN, synbiotic.

As can be seen in Figure 2, diarrhea appeared only after the first infection (day 6 with SA11), and not after the second (day 17 with EDIM), as was expected. Focusing on the former, the incidence of SA11-induced diarrhea was evaluated by two approaches whose results were very similar: the %DA (diarrheic feces in the total animals) or %DF (diarrhea in the total obtained feces). With regard to the %DA, in the DRI group it was about 27% on 1 DPI (Figure 2A); it increased up to 60% on 2 and 3 DPI, and achieved the maximum value of 87% on 4 DPI (day 10 of life). Later, on 5 DPI, it decreased to 7%, and on day 6 DPI none of the animals in the DRI group had diarrhea (Figure 2A). The inoculation of EDIM on day 17 did not induce any modification in the fecal appearance during the following days and until day 28 (Figure 2A). When the diarrhea incidence was studied in supplemented animals, all the groups showed a modulatory effect: all supplemented groups had lower %DA than the DRI group over the 3–4 DPI period (days 9 and 10 of life, *p* < 0.05). The HBC group was totally protected throughout the study period (Figure 2B).

As can be seen in Figure 2B, the PRE induced a certain %DA even before the SA11 inoculation (20–45%) and throughout the nutritional intervention period. However, the MDA in this group was 50% (Table 1), achieved on day 7 and much lower than the 87% in the DRI group (*p* < 0.01), and the %DA on days 9 and 10 (the highest in the DRI group) was reduced by up to 15 and 40%, respectively (*p* < 0.05).

**Table 1:** Clinical variables determining the diarrhea process (from day 0 to 6 DPI).

		REF	DRI	HBC	PRE	PRO	SYN
**Incidence**	**MDA**	0.00	86.67	0.00^#^	50.00^#^	35.71^#^	69.23^#^
	**MDAd**	–	10	–	7	8	8
	**daAUC**	0.00	225.00	0.00	192.50	100.00	245.80
	**daAUCn**	0.00	225.00	0.00	92.50	100.00	89.16
	**MDF**	0.00	100.00	0.00^#^	100.00	38.46^#^	81.82^#^
	**MDFd**	–	9	–	7	8	8
	**dfAUC**	0.00	368.96	0.00	376.03	122.90	425.63
	**dfAUCn**	0.00	368.96	0.00	167.50	122.90	184.89

**Duration**	**DDB**	–	8.2 ± 0.3	–	7.7 ± 0.3	8.0 ± 0.2	7.4 ± 0.4
	**DDE**	–	9.9 ± 0.1	–	11.0 ± 0.4^#^	8.8 ± 0.4^#^	10.2 ± 0.7
	**DP**	0.0 ± 0.0	2.7 ± 0.3*	0.0 ± 0.0^#^	3.6 ± 0.5*	1.1 ± 0.3*^,#^	3.5 ± 0.7*
	**DwD**	0.0 ± 0.0	2.4 ± 0.2*	0.0 ± 0.0^#^	2.2 ± 0.3*	1.0 ± 0.3*^,#^	2.9 ± 0.7*

**Severity**	**MDI**	1.05 ± 0.03	2.60 ± 0.11*	1.15 ± 0.05^#^	2.58 ± 0.11*	2.14 ± 0.22*	2.53 ± 0.19*
	**MDId**	–	8.40 ± 0.27	–	8.61 ± 0.42	8.33 ± 0.22	8.60 ± 0.54
	**sAUC**	0.03 ± 0.02	3.46 ± 0.22*	0.25 ± 0.09*^,#^	3.27 ± 0.39*	2.29 ± 0.37*^,#^	4.00 ± 0.92*
	**sAUCn**	0.03 ± 0.02	3.46 ± 0.22*	0.25 ± 0.09*^,#^	2.28 ± 0.30*^,#^	2.29 ± 0.37*^,#^	2.69 ± 0.72*

*With regard to incidence: MDA, maximum percentage of diarrheic animals; MDAd, day with maximum percentage of diarrheic animals; daAUC, area under the curve of diarrheic animals; daAUCn, normalized area under the curve of diarrheic animals; MDF, maximum percentage of diarrheic feces; MDFd, day with maximum percentage of diarrheic feces; dfAUC, area under the curve of diarrheic feces; dfAUCn, normalized area under the curve of diarrheic feces*.

*With regard to duration: DDB, day of diarrhea beginning (DPI); DDE, day of diarrhea ending (DPI); DP, diarrhea period; DwD, days with diarrhea*.

*With regard to severity: MDI, maximum diarrhea index; MDId, day of maximum diarrhea index (DPI); sAUC, area under the curve of severity; sAUCn, normalized area under the curve of severity*.

*Results are expressed as mean ± SEM (*n* = 12–21 animals/group)*.

Statistical differences: *p < 0.05 vs. REF; ^#^p < 0.05 vs. DRI.

*Groups: REF, reference; DRI, double rotavirus infected; HBC, hyperimmune bovine colostrum; PRE, prebiotic; PRO, probiotic; SYN, synbiotic*.

Figure 2B shows %DA from those groups receiving the PRO and SYN supplement. PRO only induced a clear %DA after SA11 inoculation, with this value always being lower than that of the DRI group. It should be highlighted that the MDA in this group was 36% (Table 1), achieved on day 8 and much lower than the 87% of the DRI group, and that the %DA during days 7–11 was statistically lower as well (*p* < 0.05).

The SYN induced a certain %DA before and after the SA11 inoculation in a similar way to PRE but achieved higher values (30–60%). The MDA in this group was 70% on day 8 (*p* < 0.05 vs. DRI group), and the %DA during days 9 and 10 was reduced to 23 and 30%, respectively (*p* < 0.05 vs. DRI group).

**Figure 3:**
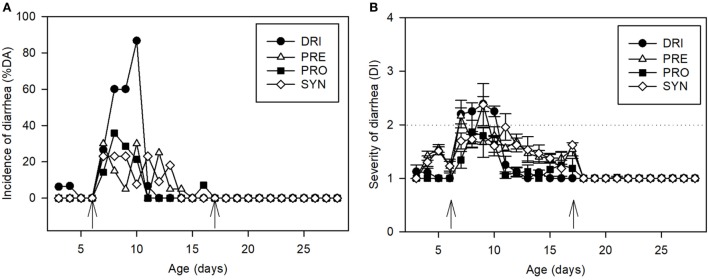
Normalized clinical indexes. **(A)** Normalized incidence of diarrhea for DRI, PRE, PRO, and SYN groups: results are expressed as % of diarrheic animals. **(B)** Normalized severity of diarrhea for DRI, PRE, PRO, and SYN groups: fecal samples are scored from 1 to 4 based on color, texture, and amount of stool. Scores on diarrhea index ≥ 2 indicate diarrheic feces. The inoculation days are indicated by arrows. Results are expressed as mean ± SEM. Statistical differences mentioned in the text (*n* = 15–21 values/group). Groups: REF, reference; DRI, double rotavirus infected; HBC, hyperimmune bovine colostrum; PRE, prebiotic; PRO, probiotic; SYN, synbiotic.

Thus, before and after the SA11 infection the PRE and SYN diets induced changes in the fecal consistency, increasing the number of feces considered diarrheic. To better dissect this direct effect, already described for certain prebiotics, a normalization of the results was performed on the basis of the average punctuation of the products obtained in the period before infection (day 3–6), and after the infection was ended (day 12–16). This procedure has been shown to be similar to that performed using data from a non-infection study (43). After normalization of the data, the effect of PRE and its combination with the probiotic in terms of incidence (both %DA and %DF) was even more evident (Table 1; Figure 3).

When the AUC of %DA was calculated (daAUC, Table 1), it could be seen that all the supplemented groups, with the exception of the SYN group, presented a lower value than the DRI group (*p* < 0.05). By contrast, the daAUC for the SYN group was higher than that in the DRI group (*p* < 0.05). However, when the daAUC was normalized by calculating the AUC of the increment of incidence during SA11 infection from the baseline of each group (without counting the non-pathogenic “diarrhea” induced by the prebiotics in the PRE and SYN groups), it was significantly lower (*p* < 0.05).

When the incidence data were calculated by using the second approach, results corresponding to the incidence of diarrheic feces (%DF) (Table 1), and their consequent parameters, i.e., MDF, MDFd, dfAUC, and dfAUCn, showed the same pattern as for the %DA.

### Duration of Diarrhea

With regard to the duration of the diarrhea process, in the DRI group diarrhea started at day 8.2 ± 0.3 (DDB) and ended at day 9.9 ± 0.1 (DDE). The DP and the DwD were 2.7 and 2.4, respectively (Table 1). The PRE and SYN diets did not modify these variables, but PRO was able to reduce both DP and DwD (*p* < 0.05) (Table 1). It should be emphasized that the PRE and SYN groups still had scores >1 until the end of the study and that these duration variables are also influenced by the direct effect on stool consistency from PRE.

### Severity of Diarrhea

An effect on stool consistency was only found after SA11 first infection, and not after EDIM infection on day 17, or a combination of both (DRI) during the second infection. With regard to the first infection, as can be seen in Figure 2C, the severity curve in the DRI group increased from day 7 (1 DPI) and was maintained at similar values until day 10 (4 DPI). At day 11, the mean score was under 2, and therefore it is not likely that the animals had diarrhea. Afterward, no animals from this group had signs of diarrhea and had a DI = 1. This diarrhea was totally prevented by the HBC intervention, as can be seen by the low punctuations obtained during the process, which were all around 1 (Figure 2C). By contrast, the PRE was not able to prevent this clinical variable of diarrhea, and the score values were similar to those from the DRI group (Figure 2D). The direct effect of this compound on fecal texture is evidenced by scores higher than 1 before infection and after the DP (days 7–12), as previously mentioned. As also observed for the incidence and duration variables, the PRO intervention was the most effective in reducing the severity of diarrhea (*p* < 0.05 vs. DRI group on days 7, 9, and 10). However, when the probiotic is administered with the PRE (SYN group) this protective action disappeared (Figure 2D). The effectiveness of the PRE and SYN diets in controlling the RV infection could not be seen through direct scoring data because the products induced features that occulted their putative action. The mean MDI for all infected groups was around 3, with the exception of that of the HBC group, which was lower (*p* < 0.05). In all cases, it was obtained around day 8 (Table 1). The AUC of the severity pattern calculated during the period with diarrhea (Table 1) showed AUC values of about 3 in inoculated animals, whereas REF animals did not develop diarrhea and had AUC values of around 0, along with the HBC group. Interestingly, a significant reduction in sAUC was observed for the PRO group with respect to the DRI group, demonstrating an overall reduction in the severity of the disease (*p* < 0.05). The PRE group only showed a significant reduction in sAUC when it was normalized (from its baseline DI present before and after the infective process), and the SYN did not modify this variable.

### Fecal Weight

**Figure 4:**
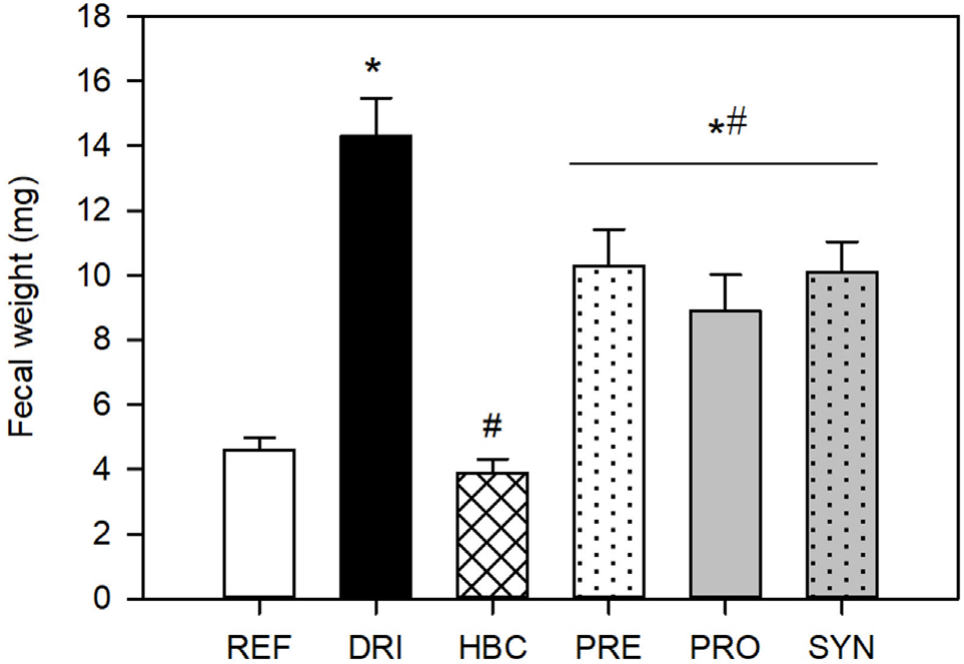
Fecal weight (mg) during the acute diarrhea period (pooled data from 1 to 4 DPI samples). Results are expressed as mean ± SEM (*n* = 32–49 samples/group). Statistical significance: *p < 0.05 vs. REF, ^#^*p* < 0.05 vs. DRI. Groups: REF, reference; DRI, double rotavirus infected; HBC, hyperimmune bovine colostrum; PRE, prebiotic; PRO, probiotic; SYN, synbiotic.

The weight of the fecal samples was recorded throughout the study. During the acute DP (1–4 DPI), animals from the DRI group had a higher fecal weight (~14 mg) than those from the REF group (~5 mg) (*p* < 0.05). The fecal weight from the HBC group in this period was significantly lower than that from the RV animals (*p* < 0.05) and similar to that from the REF group (~4 mg). The weights of fecal samples from the PRO, PRE, and SYN groups were higher than those from the REF group (*p* < 0.05), but all nutritional interventions were able to decrease the fecal weight with respect to the DRI group (*p* < 0.05) (Figure 4). Before and after this period, the fecal weight was similar among groups, and only the PRE and SYN groups had a slightly higher value (data not shown). The infection with EDIM alone on day 17 did not induce any change in fecal weight. Moreover, in all the studied groups, those double-infected without being supplemented (DRI) and those after the dietary intervention displayed a fecal weight of 27.4 ± 1.0 mg in the 1–4 days post EDIM inoculation.

### pH Changes

The pH of fecal samples from 1 to 5 DPI in each of the two infections was measured. With regard to SA11 infection, the DRI group showed a higher fecal pH (7.18 ± 0.14) on day 7 (1 DPI) than the REF group (mean 5.54 ± 0.44) (*p* < 0.05). All the nutritional interventions avoided this change and had pH values similar to that in the REF group (of about 5.5–6). The value of fecal pH one day after the second virus inoculation (EDIM on day 17) in the DRI animals (5.73 ± 0.34) was similar to those of REF or EDIM groups (5.25 ± 0.25 and 5.42 ± 0.28, respectively), and none of the diets caused significant changes in pH either (being in all cases of about 5.3–6.1).

### Temperature Changes

The rats’ body temperature was measured from 0 to 3 DPI for both SA11 and EDIM infections as a possible new marker of disease. The relative increase in temperature for the maximum value obtained after infection with respect to the 0 DPI value was calculated for each animal and is shown in Table 2. After the first infection with SA11, an increase in rectal temperature was observed among the infected animals when compared with the REF group (*p* < 0.05). Even though diarrhea was not observed in the group of animals infected only with EDIM at day 17, an increase in body temperature was found in this group (2.61 ± 0.97°C, *p* < 0.05 vs. REF group), suggesting the presence of infection. However, the presence of the first infection in the DRI animals seemed to prevent such an increase when the second infection was performed. In addition, the double-infected supplemented groups did not experience any effect on temperature after the second infection (Table 2).

**Table 2:** Body temperature increase in the peri-inoculation period (first and second infections).

	1st infection	2nd infection
REF	0.90 ± 0.29	0.21 ± 0.14
DRI	2.11 ± 0.41*	0.00 ± 0.00
HBC	4.85 ± 1.23*	1.15 ± 0.65
PRE	3.18 ± 0.95*	0.27 ± 0.18
PRO	3.42 ± 0.75*	1.02 ± 0.45
SYN	1.10 ± 0.42	0.40 ± 0.40

*Results are expressed as mean ± SEM (*n* = 12–21 animals/group)*.

Statistical differences: *p < 0.05 vs. REF.

*Groups: REF, reference; DRI, double rotavirus infected; HBC, hyperimmune bovine colostrum; PRE, prebiotic; PRO, probiotic; SYN, synbiotic*.

### Viral Shedding

In all RV-inoculated animals, the maximum viral shedding was observed on the first day after inoculation (1 DPI) for both SA11 and EDIM infections. Taking day 7 (1 DPI for SA11) into account, the HBC group had a similar RV shedding to the DRI group (101.36% with respect to the DRI group’s viral shedding). The PRO group showed a viral clearance of 135.82% compared with that of the DRI group. By contrast, the PRE and SYN groups had a lower viral shedding than the DRI group (59.33 and 57.70%, respectively, *p* < 0.05).

As regards day 18 (1 DPI for EDIM), the DRI group had lower viral shedding than that in animals only infected with EDIM at day 17, without the previous infection with SA11 (171.46% compared with the EDIM group’s viral shedding, *p* < 0.05). However, in this case, all the supplemented groups showed higher viral clearance than the DRI group (1,272.39% for the HBC group; 474.25% for the PRE group; 302.86% for the PRO group; and 434.34% for the SYN group), but without statistical significance due to the high interindividual variability found.

### DTH Response

The DTH response was studied on day 28, after 24 and 48 h post-ear priming. At 24 h, the REF group obtained an ear thickness increase of 0.86 ± 0.46. The EDIM group (without the first infection with SA11) had an increase of 1.13 ± 0.52 (*p* < 0.05 vs. REF); however, the DRI group had similar values to those obtained from the REF group (0.71 ± 0.57). None of the supplemented groups showed statistically significant differences (0.86 ± 0.70 for HBC; 1.91 ± 0.77 for PRE; 0.88 ± 0.64 for PRO; and 2.00 ± 0.77 for SYN group) with respect to the REF or DRI groups. The DTH response at 48 h followed a similar pattern.

### *Ex Vivo*-Specific Anti-RV Antibody Production

The ability of sensitized cells to produce specific Ig in the systemic (spleen) and mucosal (MLN) compartments has been studied after dietary treatment in animals only infected with SA11 or double-infected with SA11 and EDIM (Table 3). It can be seen that none of the single infections (SA11 at day 6 alone or EDIM at day 17 alone) were able to increase this variable of spontaneous ability to have natural defenses against the RV. However, the double infection induced an increase in the number of Ig-SC in the systemic compartment (*p* < 0.05). This did not happen in the mucosal compartment.

**Table 3:** *Ex vivo* anti-rotavirus Ig producing spleen and mesenteric lymph node (MLN) cells from 28-day-old animals from SA11 and double-infected (SA11 and EDIM) groups with dietary intervention.

	SA11 infection		SA11 and EDIM infections	
	Spleen	MLN	Spleen	MLN
REF	204.7 ± 40.8	245.1 ± 43.9	204.7 ± 40.8	245.1 ± 43.9
DRI	228.0 ± 21.2	318.3 ± 36.3	302.9 ± 18.5*	296.7 ± 29.3
EDIM	–	–	223.4 ± 34.6	205.5 ± 36.7
HBC	340.0 ± 75.1*^,#^	443.3 ± 43.3*^,#^	242.6 ± 51.1	215.1 ± 42.9
PRE	435.1 ± 72.1*^,#^	188.3 ± 16.4^#^	253.1 ± 23.4	246.0 ± 26.9
PRO	356.7 ± 114.0*^,#^	320.0 ± 45.8	174.6 ± 46.0^#^	216.5 ± 51.9
SYN	396.7 ± 112.7*^,#^	375.0 ± 62.1*^,#^	250.9 ± 57.3	276.2 ± 54.8

*Results are expressed as mean Ig-SC/10^6^ cells ± SEM (*n* = 3–12 animals/group)*.

Statistical differences: *p < 0.05 vs. REF; ^#^p < 0.05 vs. SA11 or DRI.

*Groups: REF, reference; DRI, double rotavirus infected; HBC, hyperimmune bovine colostrum; PRE, prebiotic; PRO, probiotic; SYN, synbiotic*.

Focusing on the first infection (Table 3), all dietary interventions induced an increase in the anti-RV Ig-SC with respect to the REF and SA11 groups, with the exception of the PRE on the MLN (*p* < 0.05 vs. SA11 group). A different pattern was found when animals were subjected to a double infection (Table 3). All dietary interventions downmodulated the Ig-SC number (*p* < 0.05) and therefore had an immune response similar to that of the non-infected group.

### *Ex Vivo* Cytokine Production

**Figure 5:**
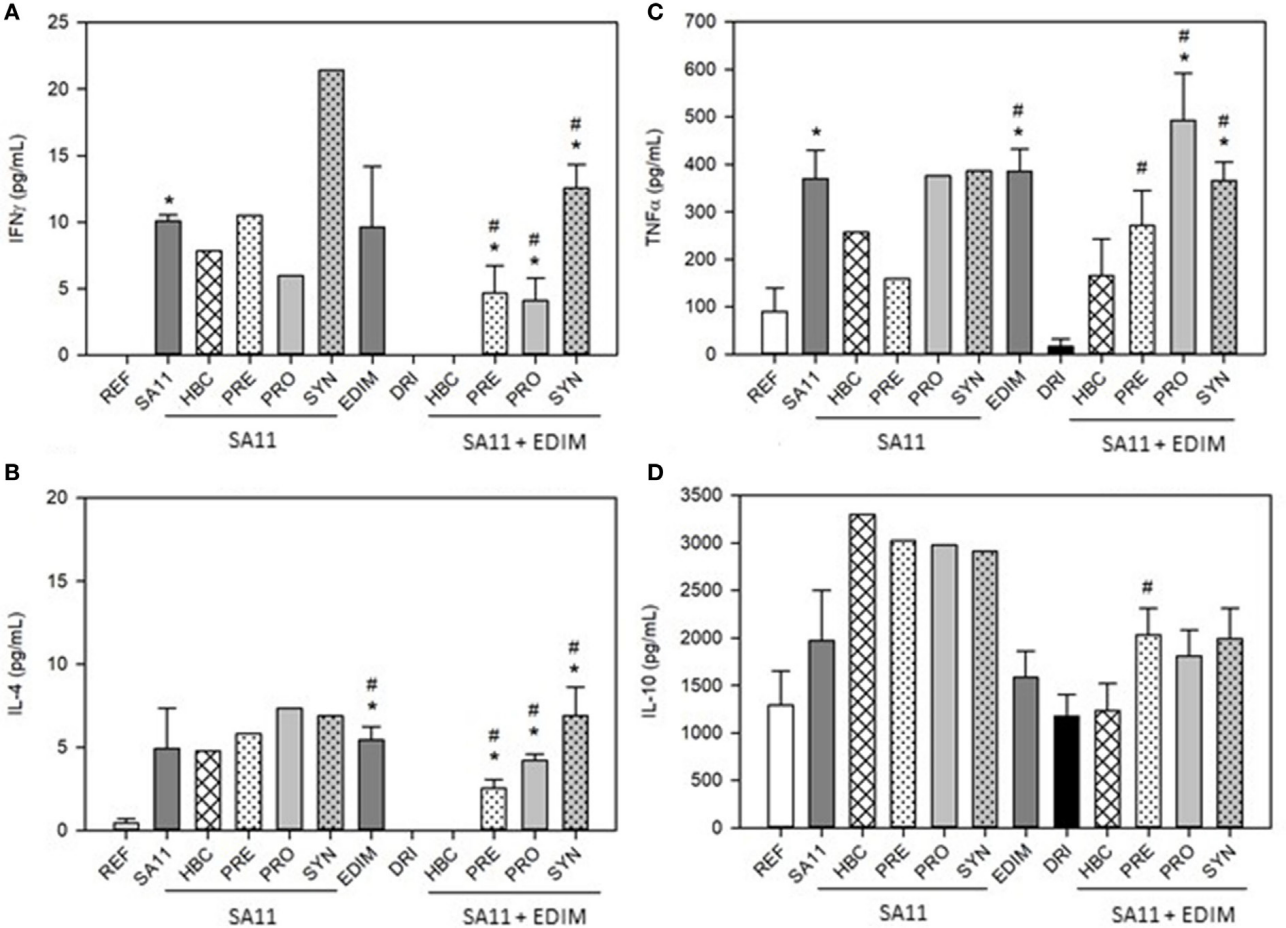
Cytokine *ex vivo* production in single- and double-infected 28-day-old animals. **(A)** IFNγ, **(B)** TNFα, **(C)** IL-4 and **(D)** IL-10 cytokine concentrations. Results are expressed as mean ± SEM (*n* = 3–12 animals/group). Statistical significance: *p < 0.05 vs. REF, ^#^*p* < 0.05 vs. SA11 or DRI (first and second infection, respectively). Groups: REF, reference; DRI, double rotavirus infected; HBC, hyperimmune bovine colostrum; PRE, prebiotic; PRO, probiotic; SYN, synbiotic.

The levels of *ex vivo* production of IFNγ by splenocytes after 72 h were under the limit of detection in some groups. This was the case for the REF, DRI, and HBC (double-infected) groups (Figure 5A). However, the groups with a single infection, either SA11 at day 6 or EDIM at day 17, had cells that were capable of responding to the challenge and produced a certain amount of IFNγ. As regards the dietary interventions in single-infected animals, none of them greatly modified the levels of IFNγ induced by SA11 in *in vitro* stimulation, with the exception of the SYN group, which had twice those levels. After the second infection, although the DRI group had no IFNγ, all the pre- and probiotic interventions induced detectable levels, with the highest effect being in the SYN group again (Figure 5A).

IL-4 was also studied (Figure 5B). This cytokine was produced at low levels in cells from the REF group and was not detected after double infection (DRI group). However, the single infections did induce IL-4, similarly to that described for IFNγ (*p* < 0.05). The pre- and probiotic supplementations did not significantly modify the IL-4 production in SA11-infected animals, although the PRO and SYN groups showed higher levels (Figure 5B). After the double infection, the DRI group had no detectable IL-4, and nor did the HBC group, but the rest of the interventions induced the production of this cytokine, with the SYN group being the one with the highest production (Figure 5B).

As for TNFα, the levels of this cytokine were low in the REF group and the infection with either SA11 or EDIM alone induced an increase of up to four times (Figure 5C, *p* < 0.05). However, this increase was not observed in the DRI group. The dietary interventions did not affect the production of TNFα when SA11 infection was performed alone, but they did induce an increase in such levels after a double infection when compared with DRI group levels (Figure 5C, *p* < 0.05).

Finally, there was no statistically significant difference between the IL-10 *ex vivo* production in the REF group, single-infected groups, and the DRI group (Figure 5D). A non-significant increase in the IL-10 production could also be seen due to the supplements in double-infected animals with respect to the DRI group.

### Mucosal IgA and Serum IgM Global Antibody Levels

The mucosal immune status was studied in the animals with different types of infections and after receiving the products at the first and second end point. The total IgA was measured in intestinal wash after the first (day 16) and second (day 28) infection. On day 16, the SA11 group had 2,367.73 ± 689.70 arbitrary units (AU) of total IgA in GW and all dietary interventions increased this value, although only the PRE and SYN groups were significantly different (7,411.41 ± 3,714.63 AU for HBC; 14,236.02 ± 3,855.70 AU for PRE; 2,998.60 ± 1,678.05 AU for PRO; and 27,800.00 ± 5,809.04 AU for the SYN group). On day 28, the DRI group displayed values of 923.94 ± 112.22 AU of total IgA in GW, which was similar to this antibody’s levels in the SA11 group at this age (1,003.60 ± 178.12), but higher than those in the EDIM group (451.90 ± 66.85, *p* < 0.05). In this case, the supplements did not affect the IgA levels, with the exception of the HBC and the PRO groups, which had lower levels (460.14 ± 97.29 AU for HBC; 1,193.12 ± 131.11 AU for PRE; 591.59 ± 96.51 AU for PRO; and 631.49 ± 142.09 AU for SYN group).

In addition, the systemic immune status in such early life was also assessed by evaluating the total IgM measurement in serum after the first (day 16) and second infection (day 28). Age influences the production of this Ig, with values on day 28 being twice as high as those at day 16 (Table S1 in Supplementary Material). On the other hand, the infections did not influence these values, and only the SYN diet was able to increase the IgM titers (*p* < 0.05) both at day 16 and day 28 with respect to the rest of the groups (Table S1 in Supplementary Material).

### Anti-RV Ig in the Intestine and Serum

Specific anti-RV antibodies were quantified in intestinal wash from 16- and 28-day-old rats as well (Table 4).

**Table 4:** Specific anti-rotavirus antibodies in gut wash (GW) (total Ig) and serum (total Ig and IgM) from 16- and 28-day-old rats.

			REF	DRI	HBC	PRE	PRO	SYN
Day 16	GW	Ig	14.85 ± 3.00	7.14 ± 2.43	6.09 ± 1.82*	13.05 ± 3.92	22.97 ± 6.94	23.45 ± 6.75
	Serum	Ig	1,624.96 ± 71.22	954.78 ± 155.66*	1,268.11 ± 195.28	1,234.32 ± 193.24	980.19 ± 68.19*	1,537.90 ± 64.49^#^
		IgM	442.64 ± 41.28	388.02 ± 61.10	540.19 ± 68.59	419.68 ± 47.22	500.79 ± 58.51	451.96 ± 99.81

Day 28	GW	Ig	27.37 ± 27.25	54.88 ± 35.31	109.08 ± 56.77	35.24 ± 23.21	71.58 ± 50.20	31.99 ± 31.82
	Serum	Ig	1,055.90 ± 81.64	1,048.51 ± 84.14	1,510.03 ± 276.22	883.89 ± 77.37	800.36 ± 64.38*^,#^	1,041.69 ± 89.86
		IgM	575.59 ± 28.71	711.26 ± 95.59	1,067.42 ± 196.08*	700.77 ± 71.57	774.38 ± 89.41	746.52 ± 75.46

*Results are expressed as mean ± SEM (*n* = 6–12 animals/group)*.

Statistical significance: *p < 0.05 vs. REF; ^#^p < 0.05 vs. DRI.

*Groups: REF, reference; DRI, double rotavirus infected; HBC, hyperimmune bovine colostrum; PRE, prebiotic; PRO, probiotic; SYN, synbiotic*.

At mucosal level, the DRI group at day 16 of life had similar, or even lower, titers of anti-RV antibodies to the REF group. Although no statistical significance was found on day 16, the pre- and probiotic supplementations showed a tendency to increase these levels. In terms of the second infection, there was an age-dependent increase in these titers, as can be seen on day 28 of life when compared with day 16, but again without significant differences between the REF and DRI groups. The group of animals only infected with EDIM had 119.27 ± 50.14 AU of anti-RV antibodies, slightly higher than that of the DRI group (with a previous RV infection). The nutritional interventions did not affect this variable either (Table 4).

Specific anti-RV antibodies were quantified in serum (total and IgM) from 16- and 28-day-old rats (Table 4). The REF group already had levels of anti-RV Ig that were even higher (*p* < 0.05) than those after SA11 infection at day 6 or EDIM infection at day 17 (682.43 ± 28.86 and 1,154.41 ± 116.44 AU, respectively). The EDIM infection alone and the DRI behaved quite similarly. Interestingly, the titers did not increase with age, and even decreased in some cases. With regard to the dietary interventions, just the PRO and SYN groups modified the anti-RV Ig (*p* < 0.05). The PRO diet downmodulated the titers at both time points, and the SYN diet up-modulated them on day 16 (Table 4).

Specific anti-RV IgM followed a similar pattern to total IgM in serum. Levels increased with age, and REF values were similar to those of infected animals, in this case with the exception of EDIM alone (905.03 ± 87.80, *p* < 0.05 vs. REF group). The HBC seemed to potentiate the production of these antibodies (*p* < 0.05 on day 28), but this was not clear with the other dietary interventions.

### Cytokine Levels in GW

**Figure 6:**
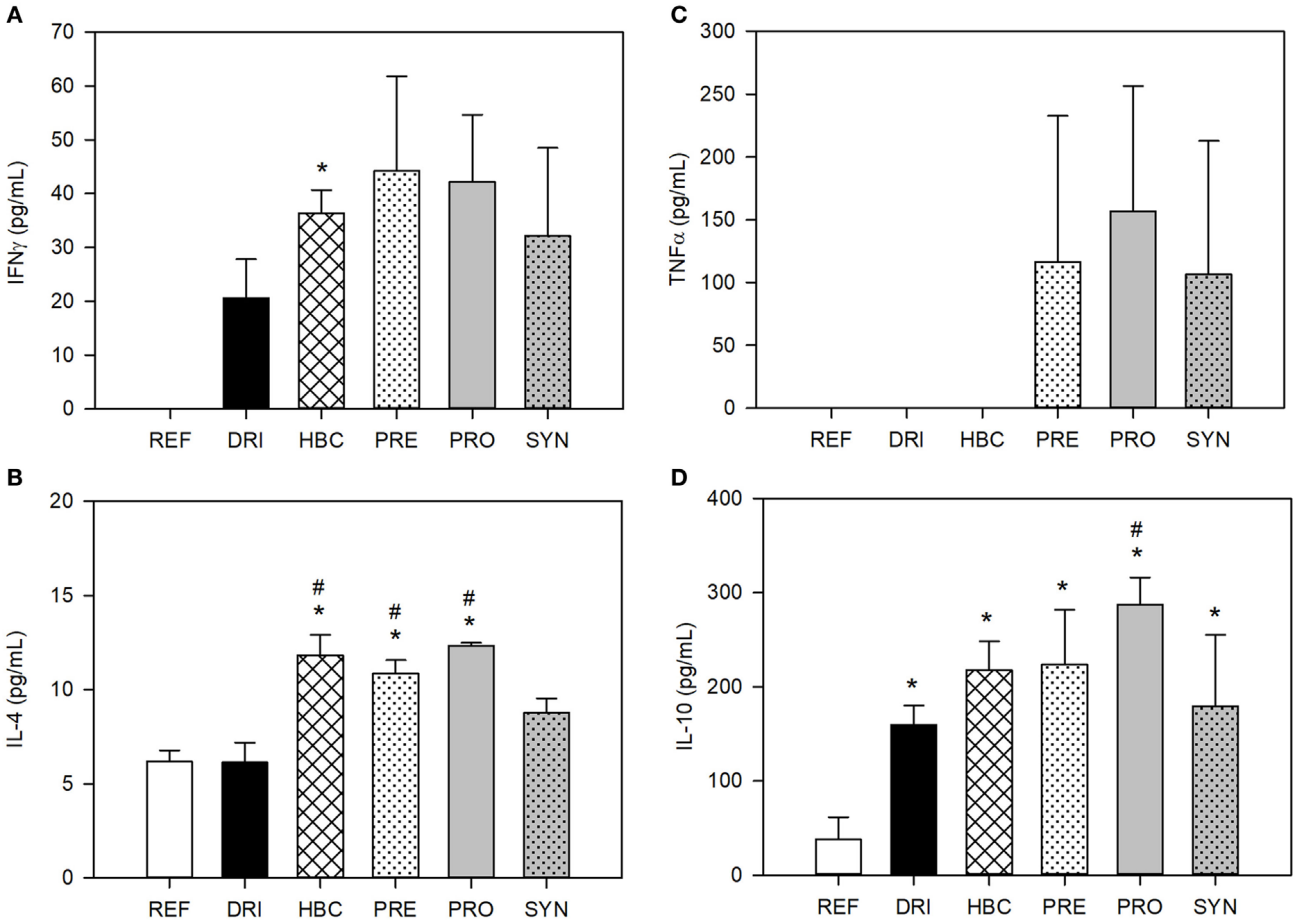
Cytokine levels in gut wash from 28-day-old rats. **(A)** IFNγ, **(B)** TNFα, **(C)** IL-4 and **(D)** IL-10 cytokine concentrations. Results are expressed as mean ± SEM (*n* = 3 animals/group). Statistical significance: *p < 0.05 vs. REF, ^#^*p* < 0.05 vs. DRI. Groups: REF, reference; DRI, double rotavirus infected; HBC, hyperimmune bovine colostrum; PRE, prebiotic; PRO, probiotic; SYN, synbiotic.

Cytokines in GW samples at the end of the study (day 28) were determined. In healthy animals (REF group), IFNγ was not detected. However, all the infected animals (double-infected with SA11 and EDIM) had quantifiable amounts of this molecule (Figure 6A). All dietary interventions also induced higher levels than the DRI animal; however, only HBC achieved statistical significance.

IL-4 was detected in all samples from the REF and infected groups (Figure 6B). Although IL-4 levels were similar in the REF and DRI groups, the animals with dietary interventions displayed higher values and were statistically different to those in the REF (*p* < 0.05) and DRI groups (*p* < 0.05), with the exception of the SYN group.

TNFα is also a cytokine measured in this fluid that was not detected in all samples. Thus far, none of the samples in the REF, DRI, and HBC groups had detectable levels. All dietary interventions with pre- or probiotics induced detectable levels of TNFα (Figure 6C).

IL-10 was found in all samples at levels ranging from 40 to 300 pg/mL. The REF group had values of ~40 pg/mL, and these values were increased by up to four times in the DRI group and even more due to the HBC treatment. All infected groups displayed significantly higher values than non-infected animals, with only those animals from the PRO group being significant vs. the DRI group (Figure 6D).

### Gene Expression Changes

The gene expression of several genes involved in immune response and intestinal barrier was studied, as can be seen in Table 5.

**Table 5:** Small intestine gene expression from 28-day-old rats.

	TLR4	Occludin	IL-10	TGFβ
REF	100.00 ± 19.46	100.00 ± 17.14	100.00 ± 17.65	100.00 ± 15.79
DRI	87.09 ± 38.38	119.48 ± 15.28	103.86 ± 28.57	101.58 ± 5.95
HBC	70.58 ± 16.75	80.13 ± 25.88	145.85 ± 47.41	106.09 ± 16.06
PRE	59.86	36.76 ± 12.00*^,#^	135.03 ± 62.54	102.54 ± 30.13
PRO	39.33 ± 7.00*	65.67 ± 12.67*^,#^	148.34 ± 74.31	62.85 ± 10.34*^,#^
SYN	39.10 ± 15.88*	50.35 ± 8.87*^,#^	92.23 ± 6.41	48.14 ± 3.16*^,#^

*Results are expressed as mean ± SEM (*n* = 6 animals/group)*.

Statistical significance: *p < 0.05 vs. REF, ^#^p < 0.05 vs. DRI.

*Groups: REF, reference; DRI, double rotavirus infected; HBC, hyperimmune bovine colostrum; PRE, prebiotic; PRO, probiotic; SYN, synbiotic*.

As regards TLRs, neither TLR2 nor TLR4 were modified by DRI or HBC interventions. However, the four dietary interventions modulated this pattern similarly. The dietary treatments with PRO or SYN significantly increased TLR2 levels (252.19 ± 62.42 and 678.41 ± 77.09, respectively) (*p* < 0.05) with respect to REF or DRI animals (100.00 ± 17.14 and 99.21 ± 55.51, respectively). By contrast, TLR4 gene expression (Table 5) was reduced by all four interventions when compared with the REF group (*p* < 0.05), suggesting a downmodulatory action on the activation of the immune response.

The analysis of IL-4 revealed that there was very low expression in this tissue and conditions (in most of the samples it was not detected and in some others it was detected with a Ct > 38). However, in the low number of samples in which it was detected the results showed that the DRI group had 143.74 ± 63.91% (compared with the 100% in the REF group), whereas the HBC and the PRO diets reduced by up to 38.33 ± 14.44 and 29.10 ± 7.23%, respectively. The gene expression of IFNγ in this intestinal tissue was even lower, with only some samples with quantifiable expression in the REF, DRI, and HBC groups, but with Ct > 39.

Occludin and claudin-2 were also evaluated and similarly to the other genes studied, the gene expression of both TJ proteins was not affected in either the DRI or the HBC animals. However, the dietary treatments induced a decrease in the occludin gene expression (*p* < 0.05) and an increase in the claudin gene expression, which was only significant in the case of the SYN group (1,115.81 ± 360.78, *p* < 0.05). Mucin gene expression was not modified by either the infective process or the dietary interventions.

Finally, IL-10, TGF-β, and foxp3 were also quantified. The gene foxp3 was not detected in any of the analyzed samples (data not shown). However, the cytokines IL-10 and TGF-β were detectable (Table 5). In the case of IL-10, its gene expression was very low and it was unaffected by the infection or the dietary supplementations. By contrast, the gene expression of TGF-β was not affected in the DRI or HBC groups, or in the PRE group, but PRO and SYN induced a significant reduction (*p* < 0.05).

### SCFA Production

**Figure 7:**
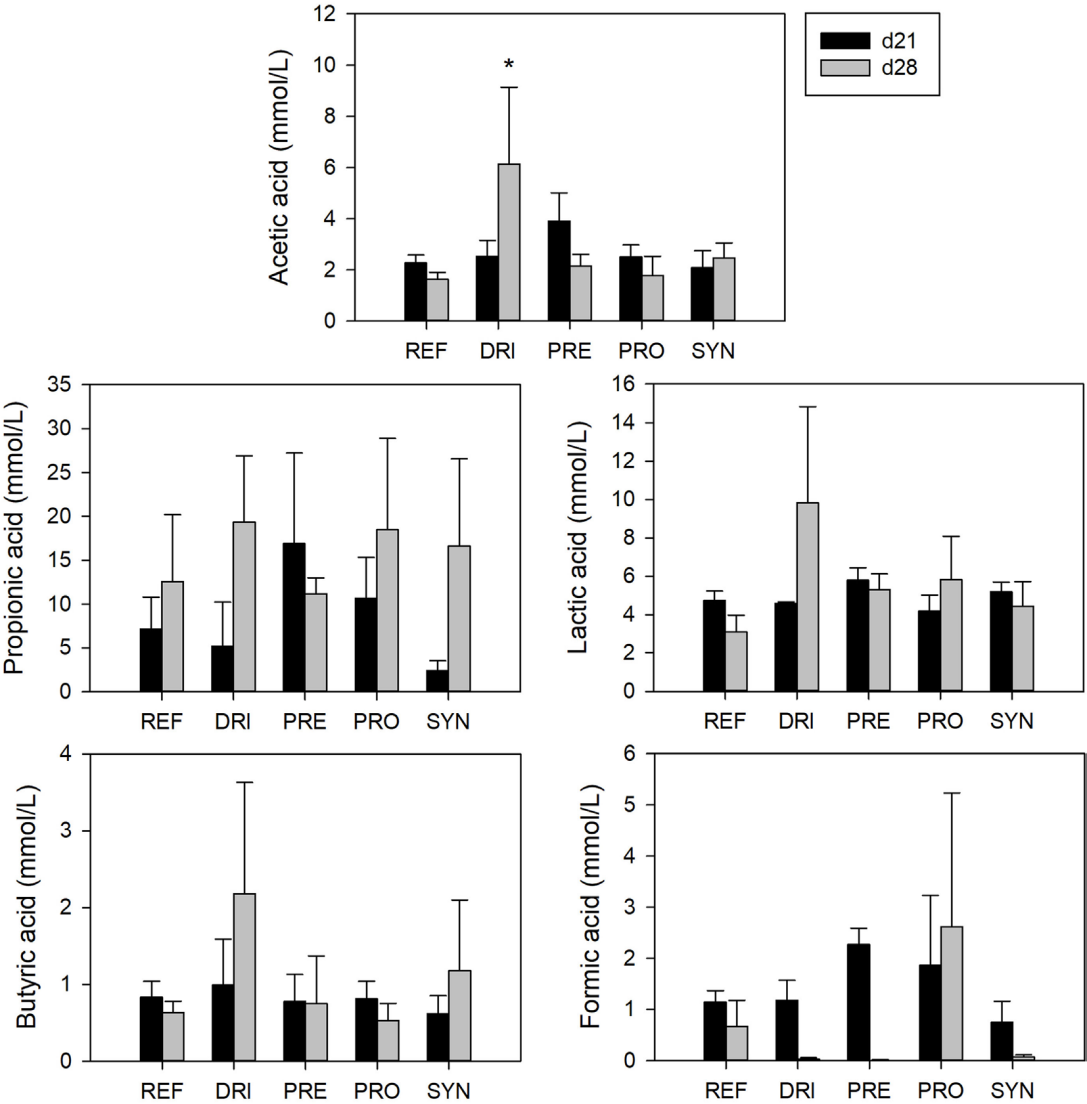
Short-chain fatty acid concentrations in feces from the different experimental groups at days 21 and 28. Results are expressed as mean ± SEM (*n* = 3–7 samples/group). Statistical differences: *p < 0.05 vs. REF. Groups: REF, reference; DRI, double rotavirus infected; HBC, hyperimmune bovine colostrum; PRE, prebiotic; PRO, probiotic; SYN, synbiotic.

The main SCFAs (acetic, propionic, and butyric), but also lactic and formic acids, were quantified in the fecal samples of 21- and 28-day-old rats. Overall, total and specific SCFAs in the REF group were not statistically modified due to RV infections or dietary interventions, except for an increase in acetic acid in the DRI group at day 28 (5.95 ± 2.72 vs. 1.83 ± 0.16 in REF, *p* < 0.05) (Figure 7).

## Discussion

Rotavirus is a major cause of acute and severe gastroenteritis in children, and although appropriate rehydration is the therapeutic intervention of choice, the introduction of other strategies such as prebiotics and probiotics is of interest (11). In this regard, by using the double-RV infection model in neonatal rats, which is more similar to the clinical reality than a simple infection model, this study has evidenced the potential of different microbial modulator products of importance in early life to modulate first infection and the host defenses for a better second infection resolution. It should be taken into account the importance of the bioactive factors present in breast milk in protecting the pups from infection. Although this nursing effect is not avoided during first infection, it was solved in the second infection by early weaning the animals the previous day of the second virus inoculation.

The model used has the appropriate features expected. Thus far, the first infection has induced mild diarrhea, which is very similar to what was found in previous studies (with the simple SA11 infection model) (23, 43, 47). The incidence, duration, and severity of diarrhea, and the fecal weight results from the DRI group after SA11 infection in this study, have been consistent with those in the RV group in previous studies (43). In addition, changes in pH and temperature have been associated with the first viral infection. Moreover, as expected, no diarrhea or changes in pH and temperature have been observed after the second infection, if the first is performed, due to the maturity of the immune system. With regard to other variables studied, the maximum viral shedding of SA11 was on 1DPI after both the first and second infections. Furthermore, a presence of specific antibodies at the systemic and at the intestinal level was found at day 14 in the previous study (43) as well as at day 16 in this one, increasing with age in both studies.

Several RV-infection animal models are already available, but most of them are single-infection models, which do not reflect the multiple reinfections that humans have during early life. Overall, although the limitation that no diarrhea is displayed in the second infection in this model, the RV double-infection rat model is suitable for studying the influence of interventions performed to regulate first infections (e.g., by vaccination, therapeutic agents, or nutritional supplementation) on the onset of a future reinfection, which so often occurs in humans. In addition, and regarding the interventional approach performed, a current limitation of this study is that the bacterial strain arrival to the gut was not studied, although it has been previously demonstrated in several studies in all humans, mice, and rats (55–57).

To evaluate the effect of modulating the first infection on the outcomes in the second viral infection a positive protection control (HBC) was used. A complete protection from diarrhea was achieved after the first infection with SA11 and it avoided the increase in fecal weight induced by RV in the acute phase of the disease. However, it increased the viral shedding after the second infection with EDIM, but still allowed the development of immune response, among other changes. These results, which are in line with those observed in other studies (25, 26), allow us to confirm its suitability as a protective control in this design.

As the second infection did not induce diarrhea we have to focus on first infection results to evaluate the effect of the prebiotic and probiotic intervention on the control of clinical symptoms. Overall, all diets seem to have a protective role somehow in some of the variables analyzed. On the one hand, the scGOS/lcFOS mixture, at the dose used here, as in previous studies (43), has a masking effect on the fecal consistency, which is one of the main limitations of this compound. This effect, also present in the SYN intervention, induced a softened stool consistency, which is not dependent on the presence of the virus. However, the scGOS/lcFOS supplementation was able to decrease the incidence and severity of diarrhea, or at least, the impact on the fecal consistency was not added to that derived from the gastroenteritis, as is observed after normalization of the results. These changes in stool consistency, bringing it closer to breastfed infants (58, 59), are thus a desirable effect. The PRO was the one with the clearest effect. *B. breve* M-16V reduced the incidence, duration, and severity of the experimental diarrhea, effects that seem to be hidden by the prebiotic addition when the synbiotic is formulated. These results are in line with those obtained in the previous study (43). In the same vein, the increase in the fecal weight seems to be a clear indicator of the incorporation of water in the total fecal content (47, 60) and therefore it has been used as an objective marker of diarrhea in this model. All nutritional interventions here avoided the fecal weight increase associated with the acute phase of diarrhea. The effects due to scGOS/LcFOS and *B. breve* M-16V are in line with those obtained in the previous study (43).

In addition, fecal pH and body temperature were measured in this study, as new non-invasive clinical markers. The fecal pH only changed during the first infection. This may be because of the electrolyte imbalances caused by the diarrhea (5). Li et al. also observed an increase in pH in the colonic content of RV-infected piglets when compared with non-infected animals (42). The dietary interventions avoided the RV-induced increase of fecal pH. On the other hand, the rectal temperature increased after the first RV inoculation with SA11 in the DRI group, but not after the second inoculation with EDIM. However, the EDIM inoculation on day 17 as a primary infection was able to induce fever on that day, so this variable shows a differential pattern between the single and the double-infection model, suggesting that the first infection allows the setting up of mechanisms involved in second infection control. Few studies have evaluated body temperature. For example, Parreño et al. measured it after the first infection of a double-infection calf model and observed fever (25). The HBC and the dietary interventions with pre- and probiotics showed a similar behavior to the DRI group, which means that, although the interventions conferred protection during the first RV inoculation, this infection was still able to allow mechanisms to control the second infection.

Viral shedding has been suggested as a marker of protection where higher presence of the virus in feces would mean higher elimination. However, this statement is not fully applicable in our model and interventions, because we have demonstrated that the scGOS/lcFOS mixture has a high capacity to bind the RV and block its infection but at the same time did not allow the virus to be detected by our ELISA technique (43). In accordance with this, the peak of viral elimination after the first inoculation with SA11 was substantially reduced in the groups supplemented with scGOS/lcFOS alone or in combination with the *B. breve* M-16V. By contrast, a similar viral shedding was observed in the group supplemented with the probiotic when compared with the DRI group after the first inoculation, as happened in the previous study (43). However, after the second inoculation, all dietary supplemented groups, even that with the scGOS/lcFOS mixture, shed a higher viral load than the DRI group, maybe as a consequence of the protection conferred during the first infection, and in line with the results obtained with HBC (26).

With regard to humoral immune response, it is described that the antibody-mediated immunity against RV involves both systemic and mucosal responses (6). This is a limitation of rodent models of RV infection because diarrhea only appears on early suckling due to the fact that their naturally acquired immune response against the virus is already highly effective in the weanling period (47, 61). In this context, the presence of specific antibodies in the model used herein did not enable clear observation of the development of protection against the virus after the first infection (although, on day 16, the animals were already weaned, the maternal influence can still be of importance on this day), or after the second infection. None of the supplementations showed a clear effect on the antibody titers either. These results contradict those obtained in the previous study (43), in which scGOS/lcFOS alone or in combination with the *B. breve* M-16V supplementation in early life increased local and systemic humoral response against the virus, suggesting a modulatory role of this intervention in the maturation of the immune system. However, some enhancing effects on the humoral immune status associated with certain dietary interventions (i.e., *B. breve* M-16V on systemic IgM) are in line with our results related to the supplementation with *B. breve* M-16V in the previous study (43) and those derived from other probiotics such as LGG or *Lactobacillus acidophilus* NCFM, which increased IgM in pigs and infants after this type of infection (62–64). In fact, a previous study was conducted to demonstrate the high immunomodulatory potential of *B. breve* M-16V in early life using the rat as a model of immune development in which the intestinal IgA synthesis was enhanced (65).

By contrast, the *ex vivo* Ig production determination allowed a differential response to be observed between the single infections and the double-infection model. The effect of the nutritional interventions could be evaluated, and they even enhanced the immune response against the first virus, while a downmodulation was observed in the second infection. This may indicate that the dietary reinforcement during the first infection at cellular level may lead to higher protection in the second one and therefore intense humoral immune response is not required. An increase in IgA-SC or IgG-SC was observed in other studies, when LGG or *L. acidophilus* NCFM were administered in pigs vaccinated and challenged with HRV (human rotavirus) (62, 64).

In terms of cellular response, several immune variables have been evaluated, such as the DTH response and the production of several cytokines. In particular, the DTH response has been described as being different depending on the model used in this context but it may reflect the primed cells before the challenge. Other studies showed a response after an infection at day 17 in mice and later suppression after a reinfection in mice who had received a primary infection before (26, 66), which are line with our study and did not allow clear conclusions to be drawn.

Focusing on cell mediators, RV triggers an immune response in the host, which is responsible for the timely resolution of the disease and the subsequent acquisition of immunity against reinfections. The cytokines produced act as mediators of immune and inflammatory responses, leading to the recruitment and activation of different populations of leukocytes, which ultimately produce cytokines in response to and against the RV. The potential part played by cytokines in the cellular response to RV has highlighted the importance of this aspect of host defense. The determination of intestinal cytokines during the peak of the diarrheic process, when it is already solved, or after *in vitro* challenge, could give an insight into the state of immune activation.

A differential pattern between the single infection and the double-infection model was also found in the *ex vivo* cytokine production, meaning that the first infection causes different immune response in the second infection. In this case, the dietary interventions enhanced the production of these cytokines not only when only one infection occurred, but also in the double-infected animals. This effect was observed for all Th1 (IFNγ), Th2 (IL-4), pro-inflammatory (TNFα), and anti-inflammatory (IL-10) cytokines. The promotion of these cytokines are in agreement with the fact that Th1 (IFN-γ)/Th2 (IL-4) cytokines initial release may participate in the inhibition of viral replication by promoting cell-mediated immunity whereas IL-10 could exert diverse roles in the pathogenicity and immunity against RV infection (67). In this line, several probiotics have shown the ability to enhance cytokine production *in vitro* (68). Wen et al. (64, 69) found an increase in IFNγ production by T-cells and a decrease in IL-10 production by Treg in pigs vaccinated with HRV and administered with LGG. On the other hand, the intestinal wash is a fluid with components from the mucosa layer that reflects the activity of the intestine. Cytokines from intestinal wash were only determined in double-infected animals and followed a similar pattern to the cytokines produced *ex vivo*.

With regard to gene expression, TLR2 and TLR4 genes were selected to be studied because TLR2 genes recognize, among other ligands, the cell-wall components such as peptidoglycan, lipoteichoic acid and lipoprotein from Gram-positive bacteria, whereas the latter bind to the bacterial lipopolysaccharide, which is the major structural component of the outer wall of all Gram-negative bacteria and a potent activator of the immune system. IFNγ and IL-4 were the selected cytokines involved in the Th1 and Th2 responses, respectively, for this assay. Besides immune molecules, others such as those from the TJ, which have been described as being modulated by probiotics *in vitro* and *in vivo* elsewhere (70), were also quantified. Mucin is a high-molecular-weight and heavily glycosylated protein produced by intestinal epithelial tissues to form a gel that acts in lubrication, cell signaling or in the formation of chemical barriers (70). Finally, molecules involved in regulatory and tolerogenic response, such as IL-10, TGF-β, and FoxP3, were also measured.

No differences in the levels of these molecules in the double-infected group (DRI) and HBC treatment were observed at day 28. This may indicate that at this age, more than 2 weeks after the second viral infection, few effects if any due to the RV infection persisted. However, the effect of the supplementation with some of the products seems to still be evident. On the one hand, the presence of prebiotics and probiotics (or both) in the gut may increase the proportion of Gram-positive bacteria (i.e., bifidobacteria and/or lactobacilli) and therefore the TLR in charge of its detection too, the TLR2. By contrast, Wang et al. (71) did not find a significant increase in TLR2 gene expression in the intestinal mononuclear cells of pigs which had been RV vaccinated and administered with LGG, but they did find an increase in TLR4 gene expression. Other studies have observed the ability of some probiotics to improve the barrier function (70), which is not clear in the present work because whereas occludin is downmodulated, claudin expression is enhanced. The results obtained here also suggest that the dietary interventions maintain or even downmodulate the regulatory and tolerogenic immune response, in disagreement with Wang et al. (71), who found an increase in IL-10 gene expression. These differences may highlight the influence of both the experimental model used and the strain studied.

Finally, the SCFA concentration found in feces was very low in all groups, which also happened in the previous study (43) and was in contrast with the increase in the SCFAs seen in other studies with GOS or GOS/FOS (72–74). This lack of positive outcome in the fecal samples analyzed as being due to the prebiotic supplementation may be a reflection of their high absorption in the colon (75), which might therefore affect their content in fecal samples.

In conclusion, scGOS/lcFOS and *B. breve* M-16V supplementation in early life is able to ameliorate RV-induced gastroenteritis whereas it allows the host to elaborate its own immune responses that would be of importance in controlling a second infection. The results obtained help in identifying key prebiotics and probiotics with modulatory effects on the maturation of defense mechanisms of the newborn, especially in the prevention and treatment of RV infections. Further studies are needed to gain a deeper insight into the effects of these compounds, and to understand if such effects could be transmittable from the rat model to humans. Moreover, the timing and dosage of administration of these microbial modulator compounds are also to be further determined. All these determinations may lead to conclusions being drawn about whether they are suitable for strengthening the mechanisms of defense of the newborn and whether this scientific knowledge generated by these results at a preclinical level could permit, at midterm the incorporation of these types of functional supplements in infant formulas.

## Ethics Statement

All experimental procedures were conducted in accordance with the institutional guidelines for the care and use of laboratory animals and were approved by the Ethical Committee for Animal Experimentation of the University of Barcelona and the Catalonia Government (CEEA-UB Ref. 493/12, DAAM: 6905), in full compliance to national legislation following the EU-Directive 2010/63/EU for the protection of animals used for scientific purposes.

## Author Contributions

FP-C, AF, MC, KL, KK, JG, and JK conceived and designed the experiments; MR-A and FP-C performed the experiments and analyzed the data, interpreted the results, and drafted the paper; all the authors contributed to the critical review and revision of the manuscript.

## Conflict of Interest Statement

The authors declare that they have a financial relationship with the organization that sponsored the research. KL, KK, JG, and JK are employees of Nutricia Research B.V. The other authors declare that they have no conflicts of interest.
