# Supplementary material for: Preventive Effect of a Synbiotic Combination of Galacto- and Fructooligosaccharides Mixture With Bifidobacterium breve M-16V in a Model of Multiple Rotavirus Infections
Source: Front Immunol. 2018 Jun 11;9:1318. doi: 10.3389/fimmu.2018.01318 (PMC6004411; doi:10.3389/fimmu.2018.01318)
Supplement: Supplementary file 1 [file table_1.docx]

**Supplementary Table 1.** Total IgM levels in serum from all groups after first and second infections. Results are expressed as mean ± SEM (n = 12–21 animals/group). Statistical significance: *p < 0.05 *vs.* REF; ^#^p < 0.05 *vs.* DRI,  ^δ^p<0.05 *vs*. the rest of the supplemented groups.

|  | **1st infection (d16)** | **2nd infection**  **(d28)** |
| --- | --- | --- |
| **REF** | 51.5 ± 2.9 | 104.5 ± 6.0 |
| **DRI** | 43.3 ± 4.1 | 101.8 ± 3.1 |
| **HBC** | 52.2 ± 5.1 | 107.9 ± 6.9 |
| **PRE** | 56.1 ± 4.8 | 112.1 ± 4.2 |
| **PRO** | 54.0 ± 3.2 | 108.0 ± 5.1 |
| **SYN** | 68.7 ± 3.9*^#δ^ | 129.5 ± 6.3*^#δ^ |
